# Management challenges in the treatment of severe hyperbilirubinemia in low- and middle-income countries: Encouraging advancements, remaining gaps, and future opportunities

**DOI:** 10.3389/fped.2023.1001141

**Published:** 2023-02-13

**Authors:** Katherine M. Satrom, Zubaida L. Farouk, Tina M. Slusher

**Affiliations:** ^1^ Department of Pediatrics, Division of Neonatology, University of Minnesota, Minneapolis, MN, United States; ^2^ Department of Pediatrics, Aminu Kano Teaching Hospital, Kano, Nigeria; ^3^ Centre for Infectious Diseases Research, Bayero University, Kano, Nigeria; ^4^ Department of Pediatrics, Global Health Program, Critical Care Division, University of Minnesota, Minneapolis, MN, United States; ^5^ Department of Pediatrics, Hennepin Healthcare, Minneapolis, MN, United States

**Keywords:** hyperbilirubinemia, neonatal jaundice, phototherapy, G6PD deficiency, low- and middle-income countries (LMIC), kernicterus

## Abstract

Neonatal jaundice (NJ) is common in newborn infants. Severe NJ (SNJ) has potentially negative neurological sequelae that are largely preventable in high resource settings if timely diagnosis and treatment are provided. Advancements in NJ care in low- and middle-income countries (LMIC) have been made over recent years, especially with respect to an emphasis on parental education about the disease and technological advancements for improved diagnosis and treatment. Challenges remain, however, due to lack of routine screening for SNJ risk factors, fragmented medical infrastructure, and lack of culturally appropriate and regionally specific treatment guidelines. This article highlights both encouraging advancements in NJ care as well as remaining gaps. Opportunities are identified for future work in eliminating the gaps in NJ care and preventing death and disability related to SNJ around the globe.

## Introduction

Neonatal jaundice (NJ) is common among all newborn infants, with as many as 80% of infants experiencing some amount of hyperbilirubinemia (1–3). Neonatal jaundice results from the accumulation of unconjugated bilirubin in the blood, due to increased production (i.e., hemolysis) and/or decreased enterohepatic clearance (4, 5). For many, the clinical course is benign and self-limited, but for some can require hospitalization and treatment with phototherapy and/or exchange blood transfusion. A subset of infants is at risk for severe neonatal jaundice (SNJ) often defined as a total serum bilirubin (TSB) level >20–25 mg/dl (6). SNJ can result in acute bilirubin encephalopathy (ABE) and chronic bilirubin encephalopathy (CBE) also referred to as Kernicterus or Kernicterus Spectrum Disorder (KSD) (7). SNJ and the resulting neurodevelopmental impairments can include a wide range of motor (choreoathetoid cerebral palsy), hearing, and cognitive challenges although intelligence is often not affected (7–9). These morbidities are largely preventable with appropriate diagnosis, treatment, and follow-up.

Long-term complications from SNJ rarely occur in high-income countries (HIC) (10–15) but remain a challenge in low- and middle-income countries (LMIC) due to lack of resources for appropriate prevention, diagnosis, and treatment of the condition (16). Accurate population-based studies are lacking, but one report based on mathematical models estimated that 1.1 million infants develop SNJ globally each year (17). A systematic review of the literature from 2017 estimated a pooled incidence of SNJ at 244 per 100,000 live births (6). These studies and others highlight the African and Asian regions having a disproportionate burden of SNJ-related disease (6, 17, 18).

There have been noteworthy improvements in the care of infants with NJ over the last years and decades. Significant effort has been made in the prevention of SNJ through parental and healthcare provider education (19, 20) and improved diagnosis with more accessible bilirubin measurement tools (21–25). The advent of phototherapy over sixty years ago (26) with improved quality of devices including the introduction to light emitting diode (LED) based units (27, 28), and subsequent upscaling and distribution of this treatment has reduced the need for exchange transfusions and therefore likely improved outcomes (29–31). Although large-scale studies are lacking, there is evidence from local LMIC institutions that demonstrate improving NJ management and outcomes. For example, the mortality rate from NJ in Taiwan decreased from 0.51% to 0.26% in the years 2000 to 2010 due to a combination of increased screening for risk factors like G6PDD, family and provider education, and improving hospital discharge follow-up. Similarly, on the Thai-Myanmar border, mortality from NJ decreased from 10% to 2% from the years 2009–2011 after the implementation of standardized guidelines and LED PT devices (32). At the Cairo University NICU in Egypt, mortality from NJ decreased from 25% in 2008 to 10% in 2015, again attributable to the combination of several interventions including protocols, training of personnel, and innovative technology (33). Lastly, NJ as a cause of neonatal death at a teaching hospital in Lagos, Nigeria, decreased from 23.3% in 2001 (34) to 11.5% in 2020 (35).

Elaboration on these improvements and others will be discussed in more detail in the coming sections. Despite these encouragements, there remain significant gaps in care across the world with SNJ as its devastating consequences disproportionately affect LMICs. These gaps highlight opportunities for improved and more equitable SNJ management (36, 37). This article based on expert cross-cultural opinion will highlight the areas of advancement in NJ care, the remaining gaps, and will suggest opportunities for future work.

## Advancements

### Prevention: emphasis on parental education

Serum bilirubin levels usually peak between three and five days after birth (5). However, most infants born in a hospital have been discharged before this peak, and many mothers in LMICs deliver at home or at a birthing center without skilled attendants (19). The American Academy of Pediatrics recommends that all newborn infants have at least one bilirubin measurement (serum or transcutaneous, TcB) before discharge (38), but this is not feasible in most LMICs due to cost and infrequent hospital births (37, 39). Therefore, the education of parents, especially mothers, can be an important aspect of the primary prevention of SNJ. This importance has been recognized in the past several years, with a few interesting developments for educational programs to empower parents to seek care in a timely way (40–42). Some strategies, highlighted in the next paragraphs, include oral presentations, pamphlets and posters, radio jingles, and the use of ictometers. Many of these educational materials can be accessed on the internet [Bilimetrixusa.org (43)].

A recent multicenter, cross-sectional study in Nigeria implemented antenatal and postnatal maternal instruction programs. This study demonstrated a decreased incidence of ABE in the group of mothers that received the intervention (maternal instruction) compared to those that received no instruction (40). This educational program, which is still in use, taught proper identification of jaundice through blanching the skin, the need to avoid hemolytic triggers due to high prevalence of glucose-6-phosphate dehydrogenase (G6PD) deficiency in the population, warnings against ineffective treatments and home remedies, and instructions on how to pursue additional evaluation and medical care for NJ (40). This instruction was delivered in multiple formats including antenatal group classes and question and answer sessions, postnatal one-on-one teaching, and written materials. This study found that delayed care-seeking for NJ was the strongest predictor of ABE and the group's maternal instruction program decreased delayed care-seeking from 49% to 17% (40).

Although general visual assessment of jaundice by family members has been encouraged in parental education efforts, by itself it is an unreliable method for detecting significant jaundice (44, 45). Icterometers can be used as a visual reference and are simple and effective tools that can be used at home by parents and at the community level by traditional birth attendants and community health workers. Harry Gosset in 1954 invented the icterometer using a Perspex glass as a non-invasive tool to assess the total bilirubin (46). It is still used as a screening tool for jaundice in LMICs. Since then, icterometers have been studied as a possible predictor of significantly elevated serum bilirubin and thus risk for SNJ (47, 48). A two-color icterometer was studied in Lagos, Nigeria, with mothers assessing the color of their newborns nose (blanched skin) and comparing to either light yellow (non-significant jaundice) vs. dark yellow (significant jaundice) and validating these assessments against either a TcB or TSB (49). This study found the two-color icterometer (Bilistrip™) to have a high sensitivity (95.8%) in detecting infants requiring phototherapy and a strong negative predictive value (91%–99%) in identifying infants at low risk of severe NJ when compared with a range of TcB thresholds (49). A similar study was conducted in China using a color card, “JCard” with eight hues of yellow, ranging from light to dark/severe and were tested on different areas of the infant's body (forehead, cheek, sternum). The cheek was found to be the most accurate location for assessment, with an area under the curve (AUC) of 0.985 when correlated with TSB > 13 mg/dl (50). Yet another example of an ictometer is the Bili-ruler which was created using advanced digital color processing and visual design to improve color matching (51). This product was developed through a collaboration between Sylhet Osamni Medical College Hospital in Bangladesh and Brigham and Women's Hospital in Boston. Its performance was similarly strong, with AUC values for identifying TcB ≥ 13 and TSB ≥ 13 mg/dl reported as 0.93 and 0.87 respectively, with an interrater reliability with 97% of measurements by two readers performing within 1 point of each other (51).

The combination of formal maternal instruction programs with simple detection tools has the potential to prevent the development of SNJ by decreasing delay in seeking appropriate medical care.

### Diagnosis: point of care bilirubin measurement

Accurate and timely measurement of bilirubin in an infant is essential to provide appropriate treatment and prevent neurological sequelae. Serum bilirubin measurement with total and direct fractions are the gold standard for diagnosing NJ but can be time intensive and require expensive equipment and trained personnel. High performance liquid chromatography is the gold standard laboratory technique, but not an option for many LMIC settings (52). Other methods used for serum measurement are Diazo reaction method and direct spectrophotometry (24, 25, 53, 54), although these still require equipment and reagents that aren't always available.

Advancements in point-of-care bilirubin determination have been very important in improving jaundice care in LMICs. TcB devices are non-invasive screening tools that have become widely used in both HIC and LMICs in the past several years. These devices use reflectance densitometry to quantify yellow skin color (21). They are user-friendly, fast, and relatively low cost. These devices are especially helpful at screening low-risk infants and determining which patients should have a serum bilirubin level measured (55). TcB is generally not recommended for use in making treatment decisions, as there can be variability among devices, different ethnic populations, gestational ages, and a limited reporting scale (56, 57). Also, the accuracy of TcB decreases with increasing TSB levels (both over and underestimation), so caution must be used and validating a high TcB (≥12–15) with a serum level is advised (58–60).

There are also emerging technologies for point-of-care (POC) devices to measure serum bilirubin. The advantages of these POC devices are their low cost, need for only a small amount of blood, the ability to use during treatment with phototherapy, and the potential to use in a field environment. Table 1 outlines the characteristics of two such POC devices (Bilistick® (22, 61) and BiliSpec (23)) and how they compare to TcB devices in general. Although these technologies hold promise to offer low-cost, accurate, and timely diagnosis of NJ, they have not been used broadly yet in LMICs partly due to concern for high failure rate in certain environmental conditions (62), questions regarding accuracy (63), paucity of testing in severe range TSB levels (23), and lack of assurance regarding long-term storage and durability.

**Table 1:** Comparison of currently available point-of-care bilirubin measurement devices.

Characteristics	Transcutaneous (56, 64, 65) (Bilicheck, BiliMed, JM-103)	BiliSpec (23)	Bilistick^®^ (22, 61)
LMIC Locations Studied	Many	Malawi	Egypt, Nigeria, Indonesia, Vietnam
Invasiveness	Non-invasive	50 μl whole blood	25 μl whole blood
Time to result	Immediate	120 s	100 s
Gold standard comparison	Clinical laboratories	UNISTAT	Clinical laboratories
Pearson's Correlation	*r* = 0.70–0.86	*r* = 0.973	*r* = 0.961
Bland-Altman (mean difference)	Varies	0.3 mg/dl	0.58 mg/dl
Calibration	Varies	Daily	Per sample
Power source	Rechargeable Battery	Battery	Rechargeable Battery
Approximate Cost (USD)	$2,000–$6,000	$150 USD Lateral flow card: $0.05	$500–$1500 Calibration kit: $250
Disadvantages	- Cannot be used to monitor response to PT - Decreased accuracy in dark skin patients and preterm infants	- Unknown performance at TSB >25 mg/dl - Long-term storage needs are unknown	- Recalibration with each sample - Need to measure sample immediately - Errors and high variability under humid conditions, high hematocrit

In settings where serum bilirubin cannot be easily or accurately assessed even by POC methods in a timely manner, the Bilirubin-Induced Neurologic Dysfunction (BIND) score is a clinical algorithm with associated web-based application (66), that has been found to predict bilirubin encephalopathy with high sensitivity and specificity (67). Although this is not a true diagnostic method, as a clinical screening tool it can be used to initiate phototherapy and exchange transfusions ahead of obtaining serum bilirubin levels in the LMIC setting if laboratory resources are immediately lacking. How widely this algorithm is currently implemented remains to be assessed. The routine use of these described methods, despite their limitations, including emerging technologies such as smartphone or artificial intelligence-based systems to be discussed later in this article, will likely improve early and prompt diagnosis of NJ.

### Treatment: phototherapy technology

Phototherapy (PT) as a treatment for NJ was first discovered over sixty years ago (26) and has since reduced the need for exchange transfusions and likely has prevented death and disability for possibly millions of neonates (29–31, 68–70). The ongoing innovation and implementation of PT in low-resource settings has improved over the recent years, making this treatment more widely available to at-risk infants in LMICs.

The main types of PT lights used in practice are halogen light sources, fluorescent tubes (regular and special), LED, and fiberoptic pads. The ideal light source has a wavelength that includes the optimal blue to blue-green range (including peak absorption for bilirubin isomerization (458 nm) (29). The PT device should have an irradiance of at least 30 *μ*W/cm^2^/nm for intensive PT (71). Halogen spotlights produce heat, which can be dangerous for the infant and thus are not preferred but are sometimes still used in LMICs. Fluorescent lights have a wavelength range of 400–520 nm with special blue fluorescent tubes offering higher irradiance. Fluorescent lights don’t produce much heat compared to halogen bulbs (72) but do lose irradiance over time and need to be monitored for efficacy regularly (73). The combination of regular (not special blue) fluorescent and white lights have been used successfully in LMICs to achieve effective irradiance levels while still being relatively low cost and accessible (74). Modifications to PT devices in LMICs such as white reflecting bassinets or blankets to optimize irradiance and exposed surface area, have become more standard practice (75, 76).

LED light sources have been shown to be effective in lowering TSB levels (77), are replacing fluorescent and halogen bulbs in high-income countries, and are becoming more widely used in LMICs as well. LEDs are advantageous because they emit a narrow bandwidth of light (450–470 nm), produce little heat, and last much longer than other types of bulbs. LED bulbs have a lifespan over double that of fluorescent lights (20,000 + h) (29). The price of these light sources has declined over recent years, making it more affordable in LMICs without the need for frequent replacements (27). Local fabrication of LED devices has been demonstrated to be low-cost and effective (28).

Lastly, innovative technology to meet the specific needs and resources of LMICs has been a welcome addition over the past several years. Filtered sunlight phototherapy (FS-PT) is one such example. Slusher and colleagues published their novel FS-PT canopy that uses commercial tinting films to remove ultraviolet and infrared light and allows multiple mother/infant dyads to sit together under the canopy (78). This method proved to be a feasible, safe, efficacious, and affordable treatment strategy for areas of the world where conventional PT is not available (79). This method is now in the process of being implemented in other LMICs such as Ethiopia, Uganda, other regions in Nigeria, and though these findings are not yet published, there is potential for future upscaling and distribution.

## Remaining gaps

### Prevention: gaps in routine screening for risk factors

The recognition of infants who are at particular risk for SNJ is paramount so that proper diagnosis and treatment can be implemented. Prematurity, postnatal bruising, and macrosomia are common postnatal risk factors that are often identified (38). One of the biggest risk factors for SNJ, however, is hemolysis, with isoimmune hemolytic disease (blood group incompatibility i.e., Rh and ABO) and G6PD deficiency being major contributors. The prevalence of Rh disease and G6PD deficiency vary among regions in the world and recognizing these important influences on SNJ requires further improvement.

Rh disease has been almost entirely prevented in HICs through universal prenatal blood type and antibody screening, Rh immunoprophylaxis, and the diagnosis and management of fetal anemia (80). The prevalence of Rh disease in these countries is estimated to be 2.5/100,000 live births, thanks to universal and coordinated perinatal care for most all pregnancies (17). In contrast, the global prevalence of Rh disease is approximately 276 per 100,000 live births, ranging widely among regions with Southeast Asia/Pacific countries at 57 and Eastern Europe at 529/100,000 live births, per 2010 data (17). This gap between HIC and LMIC care is largely attributed to the lack of routine blood group testing for both mothers and infants (which also identifies ABO incompatibility, another contributor to SNJ), as well as the high cost of immunoprophylaxis for Rh negative mothers (17, 36).

G6PD deficiency is another risk factor for hemolysis in the neonatal period and contributes substantially to the global burden of SNJ (17, 81). G6PD deficiency, an X-linked inherited condition, is the most prevalent enzyme deficiency in the world, with varying prevalence by geography and ethnicity (82). The G6PD enzyme is needed to prevent oxidative stress in the red blood cell, and thus with impaired enzymatic activity, the red blood cell is susceptible to lysis under conditions of stress (infections, medications, fava beans, traditional remedies, etc.) (82). The World Health Organization (WHO) recommends that regions of the world with a G6PD deficiency prevalence of more than 3%–5% in males should adopt universal neonatal screening and also education of healthcare workers and parents (83). Now decades later after this 1989 recommendation, routine G6PD deficiency screening programs have not been adopted in many regions, even those with a significant burden of G6PD deficiency and SNJ (81). A recent survey of healthcare providers in Nigeria, one such high risk country, illustrated gaps in knowledge of the common prevalence and need for screening of G6PD deficiency at their local hospital (20). The combination of routine screening and education of both healthcare workers and parents is needed to reduce the contribution of G6PD deficiency to SNJ globally.

### Diagnosis: gaps in infrastructure for timely diagnosis

Although there have been encouraging advancements in the diagnosis of SNJ over the past years to decades, there remains a delay in accessing care and thus timely diagnosis due to fragmented medical infrastructure. With the high rate of births happening outside of hospitals in LMICs, most infants develop SNJ at home, thus too late for any of the state-of-the-art diagnostic techniques or treatments to have full effect (37). Delays in seeking and receiving appropriate care for NJ have been reviewed as important contributors to the higher rates of SNJ in LMICs (37).

Cultural and socioeconomic barriers often contribute to a delay in diagnosis of SNJ. Families may delay seeking care due to a cultural expectation to stay home during the postpartum period, for a traditional naming ceremony, for instance (37). The advice from other family members to use traditional remedies or other treatments prior to seeking medical help can also contribute to a delay. Iskander and colleagues asked parents of infants with SNJ a series of questions to better understand reasons for late presentation to the hospital (84). None of the parents in the study received any education or follow-up instructions about NJ. Although the majority of these infants were delivered at a health care facility, once home, almost 50% of parents did not seek medical advice due to the belief that NJ was normal and self-resolving. Many families reported from this and other studies the use of herbal supplements, vitamins, antibiotics, and/or direct sunlight to treat the infant at home prior to seeking care (84–86). Once the decision has been made to seek medical attention, often the most convenient or accessible medical health center is not equipped to measure bilirubin or provide appropriate advice (37, 84). Iskander et al. reported that over 30% of parents tried 2 or 3 healthcare facilities prior to reaching the appropriate location and many traveled over 5 h to do so (84). Thus, although there have been new technologies for user-friendly bilirubin measurement and diagnosis of NJ, there remains a significant gap in actually getting the infant to a healthcare provider that has the resources to do so in a timely way.

### Treatment: need for culturally appropriate and locally specific treatment guidelines

Kernicterus happens infrequently in HICs likely due to the presence of national guidelines (71, 87, 88) and healthcare systems in place that facilitate appropriate treatment and timely follow-up (89). As mentioned above, in LMICs, healthcare is more fragmented, many infants are born at home, and arranging follow-up care is difficult (90). Because there is regional variation of risk factors of SNJ, guidelines must be locally specific to effectively prevent SNJ. Olusanya and colleagues conducted a review of existing NJ guidelines worldwide and found that very few were from LMICs and many were not high quality, according to the group's grading system (91). We do have evidence that implementing a local NJ guideline can improve outcomes, for example Thielemans et al. published a 8% decrease in mortality from SNJ after implementing a protocol at their institution (32).

Local guidelines should be specific to the region's population including prevalence of top risk factors. For example, local guidelines should recommend G6PD deficiency screening in high prevalence areas (83), unlike the US, for example, that only recommends G6PD deficiency screening when the family history of ethnic origin suggests it as a possibility or for an infant without an appropriate response to PT treatment (71). The American Academy of Pediatrics recommends pre-discharge screening for NJ with either TcB or TSB (38, 92), however this is not possible in regions where more births occur at home. In many LMIC regions, it is not common to have a routine newborn follow-up visit to assess for NJ. A neonate is typically seen during the first week of life for vaccination, however, often that visit happens after the natural bilirubin peak and other health concerns beyond vaccines are not always addressed (93). Alternative measures for follow-up that may include the role of a traditional birth attendant, midwife, or visit by a community healthcare worker with a visual screening tool, may be an alternative approach.

Local guidelines should also include reference to the use traditional remedies or practices that may delay proper treatment or lead to worsening NJ. For example, hemolytic agents, such as methylated spirits, eucalyptus oil, and henna are used in some regions to care for the umbilical cord after birth but may promote hemolysis and exaggerated NJ in infants with G6PD deficiency. To demonstrate a more direct relationship between exposure to traditional remedies and hemolysis, a zebrafish model of G6PDD was used to test the effects of traditional compounds on hemolysis. Arogbokun et al. reported that eucalyptus oil induced a 13.4% increase in hemolysis and methylated spirit showed a 39.7% increase in hemolysis in the zebrafish model (94). A survey of healthcare workers and trainees in Southwest Nigeria demonstrated that even these medical professionals often recommended hemolytic agents for the umbilical cord (82% recommended methylated spirit) (95). Other regional practices may delay proper care. For example, exposing infants to unfiltered sunlight and using antibiotics or herbal medicine are often identified in surveys of mothers when asked about care practices and beliefs related to NJ (37, 93, 96–98). Familusi and Dawodu, for example, published an association between a positive history of naphthalene exposure in the home and SNJ (need for exchange transfusion or death of the infant) (99). Because these practices vary by culture and region, they should be included in a local NJ guideline in a culturally sensitive and appropriate way.

Lastly, local treatment guidelines must include monitoring and maintenance of PT devices through routine irradiance measurements (100). Irradiance meters are used to assess the quality (dose or strength of lights) of PT delivered to infants with NJ, as the irradiance of PT devices decay with use and time (73). Florescent light bulbs decay faster than LED lights (73). In LMICs, the quality of PT is rarely monitored and a significant proportion of available PT devices may be producing suboptimal irradiances (75, 101, 102). This is because irradiance meters are expensive and not readily available (103). Recently, Powell et. al. tested an inexpensive mobile phone based irradiance meter suitable for resource constraint settings (104). Although it is ideal that the irradiance meter used it one that was specifically developed by the PT device manufacturer to measure the specific characteristics of the particular device, these are often not purchased, therefore independent low-cost meters may be a reasonable alternative. Further work into making irradiance meters affordable, accessible, and user-friendly is needed.

## Opportunities for future steps

### Prevention: emphasis on prenatal care and education

The future of prevention of SNJ needs to include an emphasis on prenatal counseling and identification of SNJ risk factors during pregnancy. Although advancements have been made in an effort to educate mothers and families as described above (40), this has largely taken place during the postpartum time frame. By identifying a fetus who is at risk for SNJ prior to birth, a birth plan can be made to ensure timely diagnosis and treatment, if needed. Delivery at a hospital or care center equipped with the laboratory technology and access to PT may be recommended so that diagnosis and treatment can be escalated in a timely and organized way. Education against hemolytic agents or other traditional remedies can take place weeks to months ahead of birth, rather than in the postpartum time when mothers may be exhausted and overwhelmed.

An emphasis on prenatal prevention must include education of the maternal providers, including obstetric physicians, traditional birth attendants, or nurse midwives. A survey of healthcare providers and trainees identified significant gaps in provider knowledge related to prevention of and possible sequela of SNJ (20). For example only 53% of healthcare providers reported seeing a case of neonatal jaundice and very few traditional birth attendants recognized risk factors for SNJ (G6PD deficiency, Rh incompatibility, preterm birth) (20). Risk factors of SNJ that should be identified during pregnancy include a family history of SNJ, Rh negative blood type, family history of G6PD deficiency or other risk factor for hemolysis.

One randomized controlled study from China demonstrated that antenatal education about jaundice led to mothers being more likely to recognize jaundice in their infants (71% in education group vs. 41% of controls) and were less likely to use traditional medicines and exposure their infants to the sun compared with control infants (105). 95% of mothers in the intervention arm knew that NJ can lead to brain damage vs. 38% of mothers in the control group. This is one example of effective antenatal counseling, although there has not been a study comparing antenatal to postnatal education, as a previously mentioned study on maternal instruction did not have an adequate sample size to compare antenatal vs. postnatal effectiveness (40).

### Diagnosis: harnessing telehealth for timely diagnosis and appropriate referral

Advancements in technology, improved access to the internet, and availability of smart phones, together can work to provide more timely and accurate diagnosis of SNJ and therefore lead to expedited treatment. The healthcare systems' recent experiences with the COVID-19 pandemic have led to more progress with telehealth in an effort to reduce in-person exposures to potential infections, when possible. As outlined previously, delayed care for neonates with SNJ is a major contributor to the development of negative outcomes. One contributor to delay is poor advice from physicians and other healthcare providers and seeking medical care at a facility that is not equipped to diagnose or treatment SNJ (19, 37).

Teleconsultation may be an efficient way to ensure proper treatment for NJ is being implemented and that a transfer to another medical facility can be coordinated if needed (106). Smartphone technology has recently been harnessed to diagnose NJ in a point-of-care manner. Taylor et al. published their BiliCam technique that used a camera with specialized software and machine learning to identify and predict bilirubin levels (107). The BiliCam had a correlation of 0.91 with TSB levels and a relatively high sensitivity (85%–100%) (107). Another study in China demonstrated decreased neonatal readmission rate and decreased maternal anxiety scores for mother-infant dyads that used a smartphone app to monitor for neonatal jaundice at home under web-based guidance of pediatricians compared to the control group that received routine care (108). Similar smartphone-based detection tools are being studied in other regions of the world, including Singapore (109), Ghana (110), and Saudi Arabia (111), and Nigeria (112), just to name a few. Although these tools are best suited as screening devices, as with the TcB, they are encouraging uses of state-of-the-art technology to improve the recognition and diagnosis of NJ. This smartphone technology could also help connect the local provider and family with a facility that is capable of next steps in care, as to avoid seeking care at a facility that is ill-equipped, thus providing efficiency in treatment.

### Treatment: improving long-term medical care for those affected by severe neonatal jaundice

Lastly, long-term medical care and support is needed to improve the quality of life for those affected by SNJ. The WHO introduced the term disability-adjusted life year (DALY) as a measure of population health taking into account the whole burden of disease (113). DALY takes into account years lost due to premature death and years of healthy life lost due to disability (113). This measurement is important to consider when it comes to disability and impairment as a result of SNJ and its neurological sequelae. For example, the DALY for SNJ would include both premature death for neonates that died due to SNJ as well as years lived with disability, including cerebral palsy, hearing loss, and cognitive challenges for survivors (18). NJ accounted for 113,401 DALYs globally in 2016 in the neonatal period and was the 15th leading cause of DALYs among children under five years of age (114, 115). One recent analysis estimated the contribution of G6PDD to SNJ and its associated burdens to be 54,251 DALYs with related economic deficits to be $309–584 million (116).

Assisted technologies such as hearing aids, cochlear implants, communication boards, eye tracking devices, and simple aids to daily living such as wheelchairs and grab bars are not readily available in many LMICs (117, 118). Early intervention with physical and occupational therapies, as early as infancy, have the potential to improve developmental opportunities for at-risk patients (117, 119). Developmental clinics with multidisciplinary care teams should be in place to follow these children over time in an organized fashion. Finally, as noted in a 2017 article in African Business “*Too many disabled Africans are excluded from the labour market. More must be done to include them”* (120). While advocacy work in Africa is increasing, much more needs to be done to assure that adults living with KSD are equipped and encouraged to contribute to their societies.

## Conclusion

In conclusion, when reviewing the past years of NJ literature, there have been encouraging progress to the way SNJ is prevented, diagnosed, and treated in LMICs. A focus on education and harnessing emerging technologies are common themes for these advancements. Remaining challenges in LMICs are related to fragmented medical care, traditional birth and postpartum practices, and the lack of locally specific and culturally appropriate management guidelines. Large scale implementation of small-scale projects noted above would be a giant step in the prevention and treatment of SNJ. Having buy-in from policy makers, as well as embracing innovative technologies such as smartphone enabled detection of NJ and filtered sunlight PT devices, should make the scourge of SNJ yet another disease relegated to the history books together with diseases such as smallpox and poliomyelitis.
