# Corrigendum: Management challenges in the treatment of severe hyperbilirubinemia in low-and middle-income countries: encouraging advancements, remaining gaps, and future opportunities

**DOI:** 10.3389/fped.2023.1181023

**Published:** 2023-05-02

**Authors:** Katherine M. Satrom, Zubaida L. Farouk, Tina M. Slusher

**Affiliations:** ^1^ Department of Pediatrics, Division of Neonatology, University of Minnesota, Minneapolis, MN, United States; ^2^ Department of Pediatrics, Aminu Kano Teaching Hospital, Kano, Nigeria; ^3^ Centre for Infectious Diseases Research, Bayero University, Kano, Nigeria; ^4^ Department of Pediatrics, Global Health Program, Critical Care Division, University of Minnesota, Minneapolis, MN, United States; ^5^ Department of Pediatrics, Hennepin Healthcare, Minneapolis, MN, United States

**Keywords:** hyperbilirubinemia, neonatal jaundice, phototherapy, G6PD deficiency, low- and middle- income countries (LMIC)

A Corrigendum on Management challenges in the treatment of severe hyperbilirubinemia in low- and middleincome countries: Encouraging advancements, remaining gaps, and future opportunities By Satrom KM, Farouk ZL and Slusher TM. (2023) *Front. Pediatr*. 11:1001141. doi: 10.3389/fped.2023.1001141

In the published article, **reference number 104** was incorrect. The reference “Powell P AI, Slusher TM, Satrom K, DeWitt G. Smartphone enabled phototherapy irradiance meter for the care of the jaundiced neonates in low-resouce regions. Frontiers in Biomedical Devices. (2020):83549. doi: 10.1115/ DMD2020-9040” has been changed to “Powell P, Abdulkadir I, Slusher TM, Satrom K, DeWitt G. Smartphone enabled phototherapy irradiance meter for the care of the jaundiced neonates in low-resouce regions. Frontiers in Biomedical Devices. (2020):83549. doi: 10.1115/DMD2020-9040”.

In the published article, there was an error in **Section 3.3** *Treatment: Need for culturally appropriate and locally specific treatment guidelines,* paragraph four. The reference to Powell et. al. in the text was misspelled.

This sentence previously stated:

“Recently, Powel et al. tested an inexpensive mobile phone based irradiance meter suitable for resource constraint settings (104).”

The corrected sentence appears below:

“Recently, Powell et al. tested an inexpensive mobile phone based irradiance meter suitable for resource constraint settings (104).”

The authors apologize for this error and state that this does not change the scientific conclusions of the article in any way. The original article has been updated.

## Publisher's note

All claims expressed in this article are solely those of the authors and do not necessarily represent those of their affiliated organizations, or those of the publisher, the editors and the reviewers. Any product that may be evaluated in this article, or claim that may be made by its manufacturer, is not guaranteed or endorsed by the publisher.

